# Evolution of liver disease severity and portal hypertension during REP 2139-Mg treatment in 3 patients with chronic hepatitis D: Case reports

**DOI:** 10.1007/s00508-026-02748-2

**Published:** 2026-06-11

**Authors:** Mathias Jachs, Michael Schwarz, Mattias Mandorfer, Andrew Vaillant, Thomas Reiberger

**Affiliations:** 1 Division of Gastroenterology and Hepatology, Department of Medicine III, Medical University of Vienna, Währinger Guertel 18–20, 1090 Vienna, Austria; 2 Vienna Hepatic Hemodynamic Lab, Department of Medicine III, Medical University of Vienna, Vienna, Austria; 3 Universitätsklinikum St. Pölten, St. Pölten, Austria; 4 Replicor Inc., Montreal, Canada; 5 Christian Doppler Lab for Portal Hypertension and Liver Fibrosis, Medical University of Vienna, Vienna, Austria; 6 CeMM Research Center for Molecular Medicine of the Austrian Academy of Sciences, Vienna, Austria

**Keywords:** HDV, HBV, Antiviral, Cirrhosis, Infectious disease

## Abstract

Treatment options for chronic hepatitis D (CHD) are limited in patients who do not respond to recommended treatment with bulevirtide ± pegylated interferon. The use of REP 2139-Mg is a novel treatment option that has recently demonstrated efficacy and safety in phase 2 trials and a compassionate access program that included three patients treated at the Vienna General Hospital with compensated advanced chronic liver disease and clinically significant portal hypertension (CSPH). Long-term outcome of CSPH patients treated with REP 2139-Mg is unknown; thus, surrogate endpoints are of interest in this population. In the 3 patients receiving REP 2139-Mg for 12 months at our center, we monitored the hepatic venous pressure gradient (HVPG) as the strongest prognostic surrogate biomarker in compensated cirrhosis at baseline, and after 3 and 12 months of treatment. The HVPG dynamics alongside additional liver function tests observed are summarized in this report and two out of three patients achieved undetectable hepatitis-D-virus(HDV)-RNA and low hepatitis B surface Antigen (HBsAg) following pronounced alanine aminotransferase (ALT) flares during REP 2139-Mg treatment. The first patient showed virologic non-response and no HVPG dynamics during treatment. The second patient developed jaundice upon treatment initiation, resulting in a transient increase in HVPG despite virologic response. The third patient achieved virologic response that was paralleled by sustained reductions in HVPG.

## Case reports

The REP 2139-Mg (Replicor Inc., Montreal, Canada) is a nucleic acid polymer blocking hepatitis B virus subviral particle assembly and hence, hepatitis D virus (HDV) replication. It has recently demonstrated antiviral effectiveness in patients with chronic HDV infection and advanced chronic liver disease (ACLD), achieving virologic response (≥ 2 log decline in HDV-RNA) and HDV-RNA undetectability in 70% and 58% of patients included in an international compassionate access program, respectively [1]. We included 3 HDV patients with previous nonresponse to pegylated interferon (PEG-IFN) and bulevirtide (BLV). As all patients had ACLD and clinically significant portal hypertension (CSPH), we measured the hepatic venous pressure gradient (HVPG) prior to and during REP 2139-Mg therapy as a strong prognostic biomarker in ACLD, reflecting portal hypertension severity that drives decompensation [2, 3]. The HVPG measurements were conducted at baseline (BL), i.e. on the day of treatment initiation before the first dose, after about 3 months of treatment (M3), and 1 week after administering the final dose of treatment, i.e., end of treatment (EOT). Simultaneously, we performed liver (LSM) and spleen (SSM) stiffness measurements [4, 5], indocyanine green (ICG) clearance [6] and a comprehensive panel of biomarkers such as interleukin‑6 reflecting systemic inflammation [7], von Willebrand factor as a surrogate for endothelial dysfunction [8, 9] or enhanced liver fibrosis (ELF) test, reflecting fibrogenesis, with higher values indicating more severe fibrosis [10].

## Patient 1

**Fig. 1:**
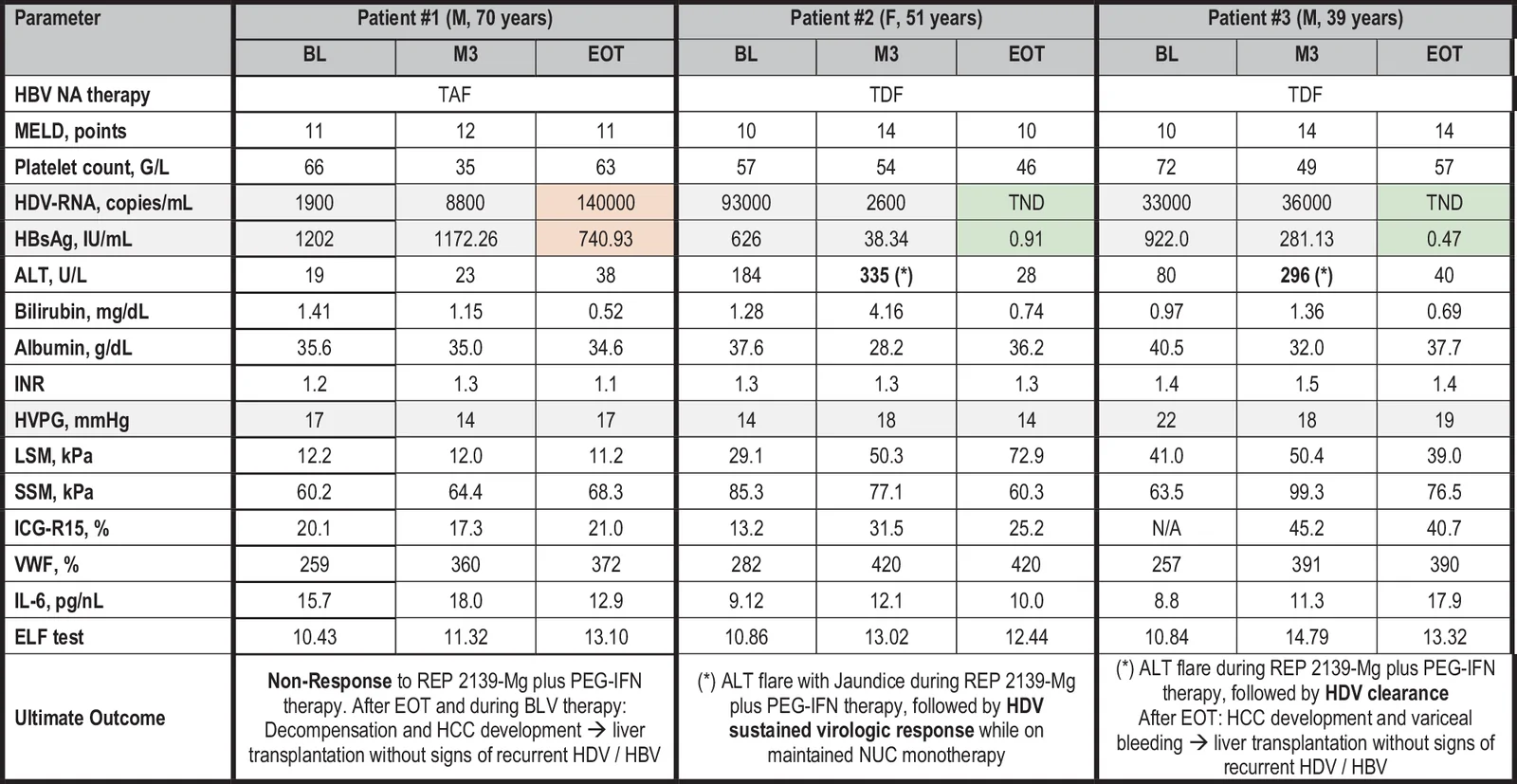
Individual evolution of patient characteristics, i.e. surrogates of viral disease activity and liver function alongside portal pressure and non-invasive diagnostic/prognostic biomarkers, during treatment in the three included patients. *TND* target not detected, *BL* baseline, *M3* month 3, *EOT* end of treatment, *HBV* hepatitis B virus, *HDV* hepatitis D virus, *TAF* tenofovir alafenamide, *TDF* tenofovir disoproxil fumarate, *MELD* model for end-stage liver disease, *HBsAg* hepatitis B surface antigen, *ALT* alanine aminotransferase, *INR* international normalized ratio, *HVPG* hepatic venous pressure gradient, *LSM* liver stiffness measurement, *SSM* spleen stiffness measurement, *ICG-R15* indocyanine green retention rate at 15 minutes, *VWF* von Willebrand factor antigen, *IL-6* interleukin 6, *ELF* enhanced liver fibrosis, *PEG-IFN* pegylated interferon alfa-2a

This 70-year-old male patient started combined treatment with REP 2139-Mg s.c. 250 mg once weekly (qW) in combination with a lower dose of PEG-IFN s.c. 90 µg qW owing to poor tolerability (pancytopenia) during previous PEG-IFN treatment. During treatment, alanine aminotransferase (ALT) was always normal, hepatitis B surface antigen (HBsAg) decreased slightly over time, while no decline in HDV-RNA was observed. Following the program protocol [1], we switched to i.v. injection of Rep-2139 Mg after 20 weeks of treatment and subsequently also increased the dose to 500 mg qW after 36 weeks of therapy. Despite these changes the patient did still not achieve a virologic response to i.v. 500 mg qW REP 2139-Mg plus PEG-IFN therapy. The HVPG slightly decreased from 17 mm Hg at BL to 14 mm Hg at M3 (14 weeks after treatment initiation) but returned to BL values at EOT (see Fig. 1). The ICG retention rate at 15 min (ICG-R15) behaved similarly to the HVPG. Of note, the results of the ELF test gradually increased during treatment. Following EOT, the patient again started BLV but again without virologic response. Half a year after EOT with REP 2139-Mg and while still on BLV, the patient developed hepatic decompensation and hepatocellular carcinoma (HCC). After locoregional bridging therapy for HCC, the patient underwent liver transplantation. The patient is currently recovering from transplantation without any signs of recurrent HBV/HDV coinfection.

## Patient 2

The 51-year-old female started combined treatment with REP 2139-Mg s.c. 250 mg qW in combination with dose-reduced PEG-IFN s.c. at 90 µg qW owing to poor tolerability (jaundice) during previous treatment. During treatment, a pronounced ALT flare was observed (peak ALT: 683 U/L at W8), accompanied by jaundice (peak bilirubin: 6.9 mg/dL at week 8 (W8); but without any increases in INR or signs of hepatic encephalopathy), necessitating PEG-IFN withdrawal. Subsequently, under REP 2139-Mg monotherapy, the ALT flare resolved and bilirubin gradually declined, reaching normal values again by W18. The HDV-RNA as well as HBsAg decreased sharply to undetectable and low levels, respectively. The patient was switched to i.v. injection of REP-2139 Mg (still without PEG-IFN) during the last 10 weeks of treatment to pursue HBsAg loss but maintained low levels of HBsAg. The HVPG was measured while the ALT flare was resolving, showing increasing values compared to BL, likely to be some degree of remaining hepatic necroinflammation. At EOT, the HVPG reached its initial BL value again. Similarly, ICG-R15 and ELF test showed a steep increase at M3 (12 weeks after treatment initiation and during the resolving ALT flare) followed by a slight decrease during continued REP 2139-Mg therapy. The patient maintained undetectable HDV-RNA and low HBsAg levels (< 1 IU/mL) following EOT without any antiviral HDV treatment, only maintaining HBV nucleoside analogue therapy with tenofovir disoproxil fumarate and has not experienced any adverse event in the following 18 months. This patient seems to have experienced a sustained virologic response.

## Patient 3

The 39-year-old male with failed previous BLV therapy started combined treatment with REP 2139-Mg s.c. 250 mg qW in combination with PEG-IFN s.c. 90 µg qW (due to severe fatigue and depression during previous PEG-IFN treatment). During combination treatment, a pronounced ALT flare was observed (peak ALT: 659 U/L at W7; peak bilirubin: 2.32 mg/dL at W12), and subsequently PEG-IFN was reduced to 45 µg qW and maintained at this reduced dose owing to fatigue for the remainder of REP 2139-Mg treatment. The ALT flare resolved over time, bilirubin returned to normal values by W15 and HDV-RNA as well as HBsAg decreased sharply to undetectable and low levels, respectively. The M3 HVPG was measured 12 weeks after treatment initiation, showing a decline in comparison to BL that was maintained at EOT. The ICG-R15 could not be assessed at BL owing to a device malfunction but showed an improvement between M3 and EOT. The ELF and LSM showed initial increases at ME (presumably still due to the ALT flare) followed by decreases observed at EOT. The patient maintained undetectable HDV-RNA und low levels of HBsAg (< 1 IU/mL) following EOT (last measurement 38 weeks after treatment), but developed hepatocellular carcinoma (HCC) diagnosed 4 weeks after EOT followed by variceal hemorrhage. The patient eventually underwent liver transplantation 9 months after EOT and has fully recovered since without any signs of recurrent HDV or HBV infection of the liver graft.

## Conclusion

Of the three patients treated with REP 2139-Mg and PEG-IFN at the Vienna General Hospital two achieved undetectable HDV-RNA and low HBsAg following pronounced ALT flares with bilirubin increases but without any signs of liver failure, while one patient did not respond to REP 2139-Mg treatment. The nonresponder showed no benefits in HVPG during treatment, while one patient who developed jaundice showed a transient increase in HVPG, returning to BL values at EOT. The second patient responded well to treatment, which was paralleled by reductions in HVPG. The ICG-R15 behaved similarly to the HVPG, while ELF test increased in both patients who went on to develop HCC during follow-up. It should be noted that HVPG measurements at later time points may have provided valuable information with respect to disease regression in the patients, although the two patients responding to REP 2139-Mg achieved HDV-RNA undetectability several months before EOT. This is evidenced by data gathered in HDV patients treated with BLV who underwent paired HVPG assessments at baseline and after 1 year of treatment [2]. In those patients, meaningful HVPG reductions were observed in almost all patients achieving virologic response; however, response times prior the second measurement varied significantly between patients and contrasting REP 2139-Mg, ALT during BLV treatment is uncommon. Overall, the fibrosis regression and HVPG dynamics in HDV may differ slightly from chronic hepatitis C infection, where pronounced, predictable and clinically meaningful decreases in HVPG where observed even 12 weeks after EOT [7].

Overall, our observations highlight that predictors for clinical benefits from REP 2139-Mg are currently lacking and that the impact on portal hypertension (HVPG) seems to depend on virologic response to treatment as previously also observed in HDV patients treated by BLV ± PEG-IFN [2].

## Data Availability

Anonymized data is available upon reasonable request to the corresponding authors.
